# *Streptococcus suis* Serotype 14: A Nonnegligible Zoonotic Population

**DOI:** 10.1155/tbed/5779652

**Published:** 2025-08-25

**Authors:** Liangliang Wu, Likuan Zhang, Xiaoqi Wang, Wei Huang, Stefan Schwarz, Andrea Brenciani, Chenglong Li, Xiang-Dang Du

**Affiliations:** ^1^Yangchun Hospital of Traditional Chinese Medicine, (The Affiliated Hospital, Guangzhou University of Traditional Chinese Medicine), Guangzhou 529500, China; ^2^International Joint Research Center of National Animal Immunology, Ministry of Education Key Laboratory for Animal Pathogens and Biosafety, College of Veterinary Medicine, Henan Agricultural University, Zhengzhou 450046, China; ^3^Institute of Microbiology and Epizootics, Centre for Infection Medicine, School of Veterinary Medicine, Freie Universität Berlin, Berlin 14163, Germany; ^4^Veterinary Centre of Resistance Research (TZR), School of Veterinary Medicine, Freie Universität Berlin, Berlin 14163, Germany; ^5^Unit of Microbiology, Department of Biomedical Sciences and Public Health, Polytechnic University of Marche Medical School, Ancona, Italy

**Keywords:** antimicrobial resistance, integrative and conjugative elements, pathogenicity, population structure, prophage, *Streptococcus suis* serotype 14, zoonotic pathogens

## Abstract

*Streptococcus suis* is an important zoonotic pathogen that can cause severe infections in both humans and pigs. The prevalence of *S. suis* serotype 14 in sporadic cases in China has been gradually increasing during recent years. However, the current understanding of *S. suis* serotype 14 is limited. In this study, we investigated the population structure, phylogenetic relationships, antimicrobial resistance, and pathogenicity of 125 *S. suis* serotype 14 strains. These strains clustered into 12 sequence types (STs) and three clonal complexes (CCs), with ST7 accounting for the highest proportion (73.6%), which indicates significant pathogenic potential, given that ST1 and ST7 are well-known high-virulence STs in *S. suis*. Bioinformatic analysis showed that all serotype 14 strains carry the virulence genes *sly* and *epf*, while 74.4% of the strains carry the virulence gene *mrp*. In the pathogenicity test (*n* = 5), the human strain Ss2301, Ss2401, and the porcine strain L966, SC42 proved to be highly virulent strains. These data highlight the virulence potential of serotype 14 *S. suis*. Tetracycline resistance genes and macrolide, lincosamide, and streptogramin B (MLS_B_) resistance genes were most frequently detected in the population. The transmission of the former genes mainly depends on integrative and conjugative elements (ICEs), while the latter depends on both ICEs and prophages. This study not only confirmed the pathogenic potential of serotype 14 *S. suis* but also provided valuable information for improving prevention and control strategies for *S. suis* infections.

## 1. Introduction

*Streptococcus suis* is a gram-positive zoonotic pathogen that usually colonizes the upper respiratory tract (especially tonsils and nasal cavity) of pigs, and occasionally also the reproductive tract and digestive tract [1]. Pigs are often represented as asymptomatic hosts for *S. suis*. However, *S. suis* infections in humans can cause purulent meningitis, sepsis, arthritis, endocarditis, and streptococcal toxic shock syndrome (STSS), of which the most common clinical manifestations are meningitis and sepsis [2, 3]. In 1968, a human case of *S. suis* infection was first reported in Denmark [4]. So far, *S. suis* has become the most common cause of adult meningitis in Vietnam and other Asian countries [5, 6].

Serotyping and multilocus sequence typing (MLST) of isolates are often used as predictors of the pathotype of *S. suis* [7]. Strains of the same sequence type (ST) can be assigned to different serovars and vice versa. To date, 29 serotypes of *S. suis* have been described [8]. It should be noted that in recent years, a new serotype designated serotype Chz and 33 new capsular polysaccharide loci (NCL) have been identified [9–12]. Serotype 2 is considered to be the most prevalent and virulent for infections of humans in most parts of the world [13]. Two outbreaks of *S. suis* in China that have attracted worldwide attention: Jiangsu in 1998 and Sichuan in 2005, respectively, both caused by strains assigned to serotype 2 and MLST type ST7 [3, 14, 15]. Therefore, most studies have focused on *S. suis* serotype 2. However, different serotypes of *S. suis* exhibit diverse characteristics in population structure and pathogenicity. Most often, human cases of *S. suis* infections are sporadic and associated with exposure to pigs or pork products. It has been reported that the prevalence of *S. suis* serotype 14 in sporadic cases in China has increased recently [16, 17]. At the same time, we also noticed that there were two cases of *S. suis* serotype 14 infection in the hospitals that we monitored. However, in the published literature, little is known about *S. suis* serotype 14 except for information from some epidemiological studies and a general study on 11 human strains of serotype 14 *S. suis* [18, 19].

The pathogenicity of *S. suis* is related to the virulence factors carried by the respective strains. The main virulence genes of *S. suis* include genes for capsular polysaccharide (*cps*), suilysin (*sly*), muramidinase-release protein (*mrp*), extracellular factor (*epf*), fibronectin-binding protein (*fbps*), and the virulence-related sequence (*orf2*). Among them, *mrp*, *epf*, and *sly* are virulence marker factors of *S. suis* [20].

In this study, a total of 125 *S. suis* serotype 14 genomes from seven different countries were analyzed, including those of two human patients and three porcine strains that we collected. The population structure, phylogenetic relationships, putative virulence-related genes, antimicrobial resistance genes, and mobile genetic elements (MGEs) were systematically analyzed by bioinformatics. In addition, animal infection experiments and antimicrobial susceptibility testing (AST) were performed on *S. suis* serotype 14 strains isolated from pigs and humans to evaluate their pathogenicity and resistance characteristics. This study is helpful to deepen the understanding of *S. suis* serotype 14 and provide valuable information for the monitoring and prevention of *S. suis* serotype 14 infections.

## 2. Materials and Methods

### 2.1. Case Descriptions

On March 1, 2023, a 46-year-old male patient who presented with fever, chills, nausea, vomiting, and weakness of one limb 2 days ago without any obvious cause was admitted the to hospital. One day earlier, the patient experienced extra frontal discomfort such as headache, body aches, and urinary incontinence. On the day of admission, the abovementioned symptoms worsened, with a maximum body temperature of 39.5°C. Contrast-enhanced computed tomography of the brain and transthoracic echocardiogram showed slight fibrotic lesions in both lungs. Further investigation revealed that the patient reported a history of right-hand finger injury 10 days ago and had a history of contact with live animals. The blood test showed an increase in white blood cells, an increase in neutrophil percentage, a decrease in lymphocyte percentage, and an increase in C-reactive protein (42.7 mg/L). Biochemical and routine findings of cerebrospinal fluid indicated low blood sugar, significant elevation of cerebrospinal fluid proteins and nucleated cells, suggesting meningitis. Subsequently, both cerebrospinal fluid and blood samples were cultured to produce α-hemolytic gray–white colonies, which were identified as *S. suis* Ss2301 by mass spectrometry and 16s rRNA analysis. According to the drug susceptibility results, intravenous infusion of vancomycin and penicillin was administered for anti-infective treatment. After 18 days of treatment, blood and cerebrospinal fluid cultures were negative, and the patient's clinical symptoms improved significantly. The patient was discharged from the hospital.

The second patient was an 80-year-old man. The patient was admitted to the hospital on August 16, 2024, with a high fever for more than 10 days before admission. The main symptoms were low back pain and high fever. The vision and hearing were normal. The patient had a history of exposure to raw pork and ate uncooked meals, but no obvious traumatic wounds were found. On August 18, 2024, *S. suis* Ss2401 was isolated from the blood sample of the patient's right foot (verified by mass spectrometry and 16s rRNA analysis). Subsequently, the patient was treated with ceftazidime injection every 12 h for four consecutive days. After the fever subsided, the patient was discharged at the request of his family.

### 2.2. Bacterial Isolates

The experimental *S. suis* strains included three swine strains, L965, L966, and SC42 preserved in our laboratory, and two human strains, Ss2301 and Ss2401, mentioned above. All strains belonged to serotype 14. Of them, L965 and L966 were isolated from the heart and joint fluid of diseased pigs with sepsis and high fever symptoms, respectively, while SC42 was obtained from nasal swabs of a healthy pig. Both L965 and L966, isolated in 2018, belonged to ST1, while SC42, isolated in 2016, Ss2301, isolated in 2023, and Ss2401, isolated in 2024, belonged to ST7. In addition, we collected the genomic information of all *S. suis* serotype 14 strains (*n* = 120) deposited in the NCBI database, which were assembled well for subsequent analysis. Strain P1/7, which was isolated from the preslaughter blood culture of a pig that died of meningitis, was used as a positive control in the infection experiment. All *S. suis* strains were cultured in brain–heart infusion (BHI) broth (Hopebio, Qingdao, China) containing 5% fetal bovine serum at 37°C.

### 2.3. Whole-Genome Sequencing (WGS) and Analysis

The whole genome DNA of *S. suis* Ss2301 and Ss2401 was extracted using the QIAamp DNA Mini Kit (Qiagen, USA) and sequenced using the MiniION nanopore and Illumina MiSeq platforms (Shanghai Personal Biotechnology Co. Ltd., China). For strains L965, L966, and SC42, only raw Illumina reads were generated. The raw Illumina reads and raw nanopore reads of *S. suis* were assembled using Unicycler (v0.5.0) [21]. The assembled sequences were annotated using RAST (https://rast.nmpdr.org/). Antimicrobial resistance genes were identified using ResFinder [22]. MLST subtyping was carried out by the PubMLST database (https://pubmlst.org/). The global optimal eBURST (goeBURST) analysis was used to analyze the possible patterns of evolutionary descent. Serotype and virulence-related genes were determined by comparison analysis after establishing a serotype library or a virulence-related genes library of *S. suis* using AbRicate. The distribution of 55 putative virulence-associated genes (including *mrp*, *sly*, *epf*) of *S. suis* was investigated among the 125 *S. suis* serotype 14 genomes, as described in previous reports (listed in Supporting Information 1 Table S1) [23]. The genes having a global match region at <80% of the amino-acid sequence with an identity of <80% were determined to be absent. The integrative and conjugative elements (ICEs) and prophages were predicted by ICEberg (https://db-mml.sjtu.edu.cn/ICEberg/) and PHAST (http://phast.wishartlab.com/), respectively.

### 2.4. Minimum Spanning Tree of *S. suis* Serotype 14 Strains

All assembled genomes of *S. suis* strains stored in the Genbank database were downloaded (*n* = 6008). The serotypes of all strains were determined by analyzing their whole genome sequences. The background information of 125 strains of *S. suis* serotype 14 is shown in Supporting Information 2 Table S2 (including five isolates from this study and 120 strains downloaded from the GenBank public database). The minimum spanning tree was constructed by BioNumerics v.7.6.

### 2.5. Phylogenetic Tree of ST7 *S. suis*

A total of 315 strains of ST7 *S. suis* were extracted from the above 6008 strains stored in the Genbank database. The background information of 318 ST7 *S. suis*, including three isolates in this study, is shown in Supporting Information 3 Table S3. A phylogenetic tree was constructed based on single-nucleotide variations. The phylogenetic tree was constructed by FastTree 2.10 and visualized by FigTree and Chiplot (https://www.chiplot.online/circleTree.html) [24–26].

### 2.6. AST

Strains L965, L966, SC42, Ss2301, and Ss2401 were selected for AST, while *Streptococcus pneumoniae* ATCC49619 as quality control. AST was performed by broth microdilution according to the recommendations given by EUCAST [27], and the results were categorized according to the EUCAST breakpoint tables for interpretation of MICs and zone diameters, version 15.0. The antimicrobial agents tested were as follows: β-lactam antibiotics (penicillin, ceftiofur, and amoxicillin), rifamycin (rifampicin), glycopeptide (vancomycin), quinolones (enrofloxacin), oxazolidone (linezolid), phenicols (chloramphenicol and florfenicol), macrolides (azithromycin and erythromycin), aminoglycosides (gentamicin, kanamycin, and spectinomycin), lincosamides (clindamycin and lincomycin) and tetracyclines (doxycycline and tetracycline). The breakpoints of *S. pneumoniae* are used for penicillin, amoxicillin, rifampicin, vancomycin, linezolid, and chloramphenicol. The breakpoints of *Staphylococcus aureus* were used for azithromycin, ceftiofur, gentamicin, kanamycin, and spectinomycin. The breakpoints of *Streptococcus dysgalactiae* were used for enrofloxacin and lincomycin.

### 2.7. Infection Experiments

The pathogenicity of strains L965, L966, SC42, Ss2301, and Ss2401 was determined by intraperitoneal injection of the tested strain into mice. 6-week-old female BALB/c mice were purchased from Liaoning Changsheng Biotechnology Co., Ltd. Before infection, each strain was collected in the mid-log phase, washed twice with PBS, and adjusted to the appropriate doses in PBS for infection. Ten mice in each experimental group were intraperitoneally injected with 3 × 10^8^ CFU per mouse. The final mortality of mice was calculated after 96 h of continuous observation after infection. Serotype 2 *S. suis* P1/7 served as a positive control due to its high pathogenicity and known characteristics, and the PBS group was used as a negative control [28, 29]. According to previous reports, strains with a mortality rate of ≥80% in mice are defined as highly virulent strains [16, 30–32]. The experiment was conducted independently at least three times for each strain. The survival curve of each strain and the survival curve of the highly virulent strain P1/7 were statistically analyzed using the Mantel–Cox test.

## 3. Results

### 3.1. Serotype and MLST Typing

**Figure 1:**
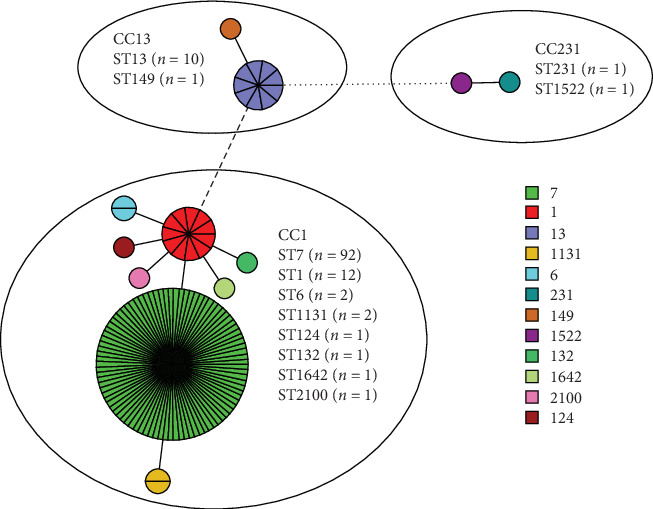
The minimum spanning tree of MLST data of serotype 14 *S. suis* strains (five strains from this study and 120 strains extracted from GenBank) was constructed. Each node represents a unique ST, and the size of the circle is proportional to the number of strains with the same ST. Different ST types are represented by different colors; the difference of one allele was represented by a thick solid line, the difference of three alleles was indicated by dotted line, and the difference of more than three alleles was indicated by stippled line. Different ellipses represent different CC types, and different STs and numbers are marked inside the ellipses.

In order to investigate the molecular characteristics of the serotype 14 strains, five strains in this study and all the assembled 6008 *S. suis* strains stored in the Genbank database were included in the statistics. Both serotypes and MLST types were determined. Among the 6013 *S. suis* strains, the prevalence of serotype 14 was 2.08% (125/6013): among them, 77.6% (97/125) were from China, followed by 10.4% (13/125) from the Netherlands. 23.2% of the serotype 14 isolates were from pigs (29/125), 76% of the serotype 14 isolates were from humans (95/125), and the source information of the remaining strains was lost. The time span was from 1960 to 2024. It is worth noting that the 125 serotype 14 strains can be divided into 12 STs, of which ST7 accounted for the highest proportion of 73.6% (92/125), followed by ST1, accounting for 9.6% (12/125), and ST13 accounting for 8% (10/125). The remaining STs were only detected in one or two strains (Supporting Information 2 Table S2). 12 different STs were assigned to three clonal complexes (CCs), including CC1 (ST7, ST1, ST6, ST1131, ST124, ST132, ST1642, ST2100), CC13 (ST13, ST149), and CC231 (ST231, ST1522) (Figure 1).

### 3.2. Minimum Spanning Tree and Phylogenetic Tree Analysis

**Figure 2:**
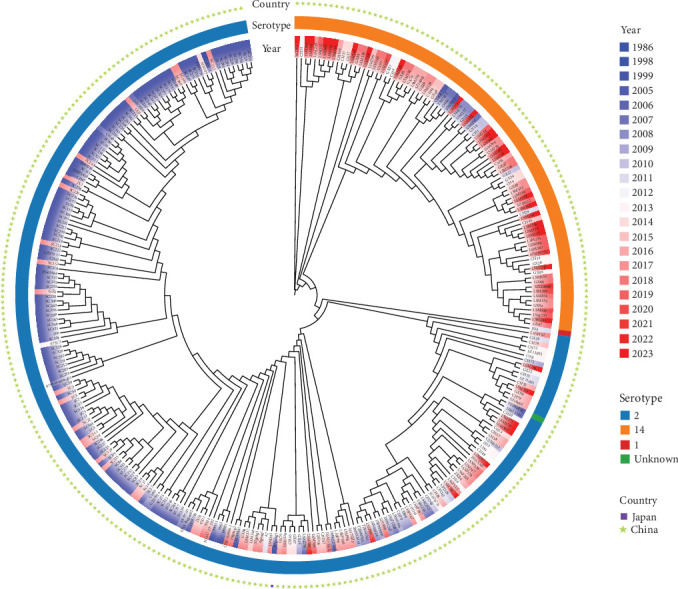
Phylogenetic tree of *S. suis* ST7 strains. The maximum likelihood phylogenetic tree was constructed using the core genomic SNPs of 318 *S. suis* ST7 strains (three strains from this study and 315 strains extracted from GenBank). The innermost circle indicates the different years of isolation of the strains, which are represented by different colors. The middle circle indicated the serotypes of all strains: blue represented serotype 2, orange represented serotype 14, red represented serotype 1, and green represented an unknown serotype. The outermost circle indicates the geographical isolation site of the strains: green asterisks represent China, and the purple square represents Japan.

A total of 6008 assembled genomes of *S. suis* were stored in the Genbank database, and all serotype 14 strains were extracted from the global 6008 assembled genomes of *S. suis*, which were used to construct the minimum spanning tree. From the perspective of the minimum spanning tree branch, ST1 occupies the core position and ST7 dominates in number, which accounts for 73.6% of all strains (Figure 1). The high detection rate of ST7 strains suggests significant pathogenic potential. In view of the large number of ST7 strains, subsequently a SNP-based phylogenetic tree of ST7 strains was constructed in order to understand the phylogeny of ST7 strains. All ST7 strains were extracted from the global 6008 assembled genomes of *S. suis* and the three genomes of this study. 318 nonrepetitive ST7 *S. suis* strains (5.29%; 318/6013) were selected to construct a phylogenetic tree. Among them, 317 strains were from China, isolated during 1998–2023, and only one strain was from Japan in 1986 (Figure 2). Among ST7 strains, serotype 2 accounted for the largest proportion (71.07%; 226/318), followed by serotype 14 (28.3%; 90/318).

### 3.3. Distribution of Putative *S. suis* Virulence-Related Genes

**Figure 3:**
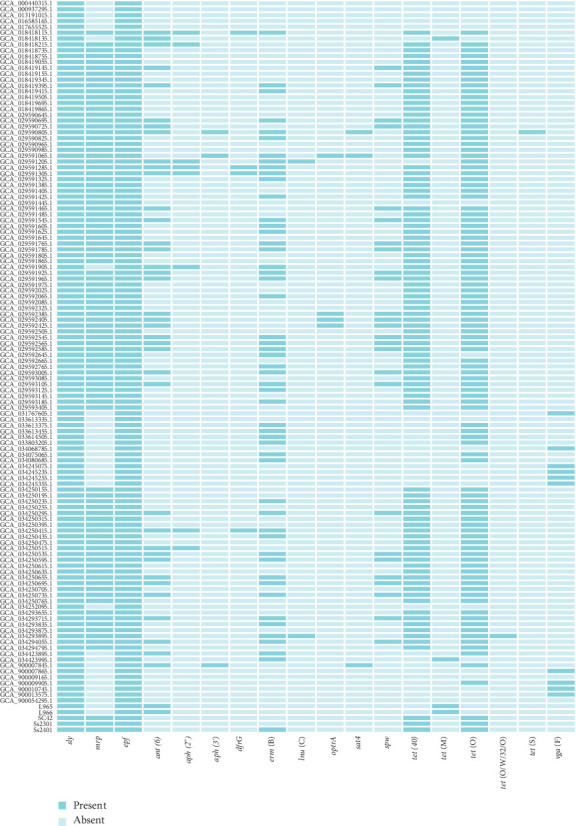
Distribution of resistance genes and three major virulence genes among *S. suis* serotype 14 strains. The vertical axis represents the strain names, and the horizontal axis represents the resistance genes or the three major virulence genes. The grid intersecting the horizontal and vertical axes is dark petrol, indicating that the strain carries the gene, and light petrol indicates that the strain does not carry the gene.

In order to identify the virulence genes carried by serotype 14 *S. suis*, 55 putative virulence factors, including three classic virulence genes *sly*, *mrp*, and *epf*, were scanned by the abricate software. The distribution of all virulence genes is shown in Supporting Information 1 Table S1, while only the distribution of the three classic virulence genes is shown in Figure 3. The results showed that different strains carry 45–50 putative virulence genes, with 68.8% of strains (86/125) carrying 50 virulence genes, followed by 28% of strains (35/125) carrying 49 virulence genes. We did not observe differences among the strains of different STs. It is worth noting that all serotype 14 strains carried the virulence genes *sly* and *epf*, while 74.4% (93/125) of the strains carried the virulence gene *mrp*. The high prevalence of the virulence genes in serotype 14 strains suggests that serotype 14 *S. suis* may have a substantial virulence potential.

### 3.4. Distribution of Antimicrobial Resistance Genes in *S. suis* Serotype 14 Genomes

In order to identify the antimicrobial resistance determinants carried by serotype 14 *S. suis*, ResFinder software was used to determine the drug resistance genes carried by all strains. Among the 125 strains, 10 strains did not carry drug resistance genes, accounting for 8%, while the remaining strains carried one to seven drug resistance genes. The distribution of antimicrobial resistance genes in 125 strains is shown in Figure 3. The resistance genes carried by *S. suis* serotype 14 mainly include seven types. A total of 41 strains carried aminoglycoside resistance genes, including *ant*(6′) encoding an aminoglycoside nucleotidyltransferase, *aph*(2″), *aph*(3′) encoding aminoglycoside phosphotransferases, and *spw* mediating spectinomycin resistance, accounting for 32% (40/125), 6.4% (8/125), 2.4% (3/125), and 20.8% (26/125), respectively. In total, 106 strains carried tetracycline resistance genes, mainly including *tet*(40) and *tet*(O), accounting for 73.6% (92/125) and 80.8% (101/125), respectively. The genes *tet*(O/W/32/O) and *tet*(S) were only detected in one strain, and *tet*(M) was detected in four strains. The macrolide, lincosamide, and streptogramin B (MLS_B_) resistance gene *erm*(B), encoding an rRNA methylase, was present in 41.6% (52/125) and the lincosamide resistance gene *lnu*(C), gene encoding a lincosamide nucleotidyltransferase, was detected in 1.6% (2/125) of the strains, respectively. Three and 10 strains carried the streptothricin resistance gene *sat4* and the streptogramin A resistance gene *vga*(F), respectively. The detection rate of the oxazolidinone/phenicol resistance gene *optrA* was 3.2% (4/125). In addition, four strains carried the dihydrofolate reductase gene *dfrG*, which confers trimethoprim resistance. In summary, tetracycline resistance genes were most commonly found in serotype 14 *S. suis*, followed by the MLS resistance genes.

In order to further understand the potential spread of resistance genes in *S. suis* serotype 14 strains, the location of the resistance genes was analyzed by whole-genome sequence analysis. Tetracycline resistance genes *tet*(O) and *tet*(40) are co-localized on an ICE, which is frequently inserted into the 3-end of the *rumA* chromosome gene and produces imperfect target repeats of 20 bp at both ends (CACGTGGAGTGCGTAGTGTT [attL]; TTCTCAAGGAACAGACTACA [attR]). It is worth noting that almost all *tet*(O) and *tet*(40) genes are located on the integrative and mobilizable elements (IMEs) inserted into the SNF2 family protein, subsequently integrated onto ICEs. In addition, there are three kinds of vectors for the MLS_B_ resistance gene *erm*(B): (i) the *erm*(B) gene alone is located on a prophage; (ii) the *erm*(B) gene may be co-localized with aminoglycoside resistance genes [*ant*(6′), *aph*(2″), etc.] on ICEs or prophages; (iii) the *erm*(B) gene may be co-localized with tetracycline resistance genes [*tet*(O), *tet*(40), etc.] on ICEs. Thus, in *S. suis* serotype 14, the main vehicles for the spread of tetracycline resistance genes are ICEs, while the spread of *erm*(B) genes is the result of the combined action of ICEs and prophages. These data reveal the potential reasons for the high prevalence of tetracycline resistance genes and the MLS_B_ resistance gene *erm*(B).

### 3.5. Antimicrobial Susceptibility Profiles of Available Strains

In order to determine the antimicrobial susceptibility of *S. suis* serotype 14, AST was performed on available serotype 14 strains. The susceptibility of 18 antibiotics in 10 categories was detected, including β-lactam antibiotics (penicillin, ceftiofur and amoxicillin), rifamycin (rifampicin), glycopeptide (vancomycin), quinolones (enrofloxacin), oxazolidone (linezolid), phenicols (chloramphenicol and florfenicol), macrolides (azithromycin and erythromycin), aminoglycosides (gentamicin, kanamycin and spectinomycin), lincosamides (clindamycin and lincomycin) and tetracyclines (doxycycline and tetracycline). All tested strains were resistant to tetracycline, while Ss2401 was additionally resistant to erythromycin, azithromycin, and clindamycin (Table 1). The whole genome sequence analysis of the tested strains showed that L965 and L966 carried the resistance genes *tet*(M) and *tet*(40); SC42 and Ss2301 carried resistance genes *tet*(O) and *tet*(40); Ss2401 carried resistance genes *erm*(B), *tet*(O) and *tet*(40), which is in agreement with the results of phenotypic AST.

### 3.6. Pathogenicity of the Serotype 14 Strains

To evaluate the virulence of the selected *S. suis* serotype 14 strains, we conducted mouse pathogenicity tests. As shown in Table 2, the mortality rate of all strains fluctuates between 60% and 100%, highlighting the significant threat posed by serotype 14 *S. suis*. Among the tested strains, except for strain L965, the other four strains had a mortality rate of over 80% in mice and were defined as highly virulent strains regardless of their origin, indicating that strains derived from healthy pigs also need to be investigated. There have been similar reports before [16, 30]. It is worth noting that L966, Ss2301, and Ss2401 exhibited virulence levels almost identical to P1/7 within 12 h after infecting mice. L965 and L966 belong to the ST1 type, while SC42, Ss2301, and Ss2401 belong to the ST7 type. However, no difference in mortality rate was observed among different ST strains.

## 4. Discussion

Human cases of *S. suis* are mainly related to serotypes 2 and 14 [33]. Previous studies reported that serotype 14 is one of the most common serotypes in human infection cases, second only to serotype 2 [17, 34–36]. In a study of human isolates of ST7 *S. suis* in Shenzhen, China, from 2005 to 2021, serotype 14 isolates were isolated from a patient who subsequently died of STSS [8]. Thailand has reported the most cases of serotype 14 *S. suis* in humans or pigs. In 2020, a study on the carriage rates of *S. suis* in pigs in slaughterhouses in Phayao Province, Thailand, showed that the carriage rates of serotypes 2 and 14 were 6.7% and 2.6%, respectively [35]. Another study on the clonal transmission of human *S. suis* serotype 14 showed that 12 (6.8%) of 177 human isolates in Thailand were identified as serotype 14, which is becoming a more common cause of human infections in Thailand [17]. In addition, a recent study from Thailand revealed that the pathogenic potential of virulent serotype 14-ST105 strains, which reduced adhesion and invasion to two epithelial cell lines (A549 and HeLa) [19]. In this study, 125 strains of *S. suis* serotype 14 were divided into 12 STs, mainly ST7, accounting for 73.6% (92/125). Therefore, we constructed a phylogenetic tree of the global ST7 *S. suis*, and the data showed the evolutionary relationship and genetic differences of ST7 strains. As many of the confirmed infections involve humans, these data indicate that *S. suis* serotype 14 is a potential zoonotic pathogen.

*S. suis* is also a reservoir of clinically important antimicrobial resistance genes [37–39]. The prevalence of antimicrobial resistance genes in this study is consistent with related reports: *tet*(O) and *erm*(B) are the most common genes associated with tetracycline and MLS_B_ resistance in *S. suis* [40, 41]. ICEs and prophages play a crucial role in the transmission of antimicrobial resistance genes in *S. suis*, but major vehicles may differ for different serotypes [42]. For example, in serotype 4 strains, prophages are the main vector for the spread of resistance genes and play a crucial role in the spread of the MLS_B_, tetracycline, aminoglycoside, and oxazolidinone resistance genes [32]. In *S. suis* serotype 14, ICEs and prophages are the carriers of tetracycline resistance genes, and the spread of *erm*(B) genes is the result of either ICEs or prophages. In view of the limitations of the available serotype 14 strains, AST of the five strains determined in this study showed that all the tested strains were resistant to tetracycline, while Ss2401 was also resistant to erythromycin and clindamycin. Due to the dependence of both humans and pigs on antimicrobial agents for the treatment of *S. suis* infections, monitoring antimicrobial susceptibility will provide valuable information for the clinical treatment of *S. suis* infections.

The pathogenicity of *S. suis* is related to the virulence factors it carries. There are a number of virulence factors of *S. suis* have been detected, including *cps*, *sly*, *mrp*, *epf*, *fbps*, *orf2*, etc. In this study, 55 putative virulence factors were detected, with a high detection rate in serotype 14 *S. suis*. Among them, *mrp*, *epf*, and *sly*, are virulence marker factors of *S. suis* [20]. Sly can utilize host cell polysaccharides as high-affinity cell receptors and may play an important role in immune escape [14, 43]. Mrp may have an impact on crossing the blood–brain barrier and attachment to host cells, and also helps *S. suis* to resist phagocytosis by phagocytes and the killing effect of polynuclear neutrophils [44]. Epf can enhance bacterial infection, invasion, and pathogenicity, thereby facilitating bacterial colonization [45]. In this study, these three classical virulence markers have a high prevalence in *S. suis* serotype 14, indicating that *S. suis* serotype 14 may have great virulence potential. It is worth noting that among the four strains defined as highly virulent strains in mouse pathogenicity experiments, three strains (human strains Ss2301, Ss2401, and healthy pig strain SC42) have virulence genotypes of *mrp*^*+*^*epf*^*+*^*sly*^*+*^. These data indicate that strains carrying virulence genes *mrp*, *epf*, and *sly* simultaneously may have stronger virulence. Further studies are needed to determine the virulence markers of serotype 14 strains. According to reports, healthy pigs are a reservoir of strains with high virulence potential for humans [46, 47]. Interestingly, in the pathogenicity test, the strain SC42 from healthy pigs was classified as a highly virulent strain, while the strain L965 from diseased pigs was classified as a strain with reduced virulence, indicating that there was no correlation between the virulence level of the strain and its source (diseased pigs or healthy pigs). Since the clinical symptoms of pigs may also depend on the co-infection with certain viral and bacterial pathogens, it may not be possible to accurately assess the public health significance of a strain based solely on the clinical information of its host.

In summary, this study clarified the population structure, antimicrobial resistance, pathogenicity, and MGEs of *S. suis* serotype 14 by the construction of a phylogenetic tree, WGS, AST, and pathogenicity tests. The results showed that the *S. suis* serotype 14 had an obvious population structure, which consisted of a variety of ST types, of which ST7 dominated. The tetracycline and MLS_B_ resistance genes-carrying ICEs and prophages jointly promoted the circulation of these resistance genes within the *S. suis* serotype 14 population. This population has a variable pathogenicity potential, with the virulence genes *mrp*, *epf*, and *sly* simultaneously harbored by more virulent strains. Since the virulence of *S. suis* is multifactorial, involving various virulence factors and their interactions, further studies are needed to determine the virulence markers of serotype 14 strains. Our data confirmed that *S. suis* serotype 14 is a nonnegligible pathogen, deepened the understanding of the *S. suis* serotype 14 population, and provided valuable information for improving the prevention and control strategies for *S. suis* infections.

## Figures and Tables

**Table 1:** Susceptibility results of the test strains.

Strains	MICs (mg/L)																	
	PEN	CEF	AMO	RIF	VAN	ENR	LZD	CHL	FFC	AZM	ERY	GEN	KM	SPC	CLI	LIN	DOX	TET
L965	0.25	<1	<1	<1	<0.25	<1	<1	2	<1	<1	<1	8	32	64	<0.25	<1	4	32
L966	0.25	<1	<1	<1	<0.25	<1	<1	2	<1	<1	<1	8	8	64	<0.25	2	4	32
SC42	0.25	<1	<1	<1	<0.25	<1	<1	<1	<1	<1	<1	2	4	16	<0.25	<1	2	16
Ss2301	0.25	<1	<1	<1	<0.25	<1	<1	2	<1	<1	<1	8	64	64	<0.25	<1	4	32
Ss2401	0.25	<1	<1	<1	<0.25	<1	<1	4	<1	64	>512	32	16	64	512	32	2	64
ATCC49619	0.25	<1	<1	<1	<0.25	<1	2	2	2	<1	<0.25	2	4	64	<0.25	<1	<1	<1

Abbreviations: AMO, amoxillin; AZM, azithromycin; CEF, ceftiofur; CHL, chloramphenicol; CLI, clindamycin; DOX, doxycycline; ENR, enrofloxacin; ERY, erythromycin; FFC, florfenicol; GEN, gentamicin; KM, kanamycin; LIN, lincomycin; LZD, linezolid; PEN, penicillin; RIF, rifampin; SPC, spectinomycin; TET, tetracycline; VAN, vancomycin.

**Table 2:** The mortality rates of mice injected with *S. suis*.

Strains	Source	Death in each period								Total deaths	Mortality rate (%)	*p*	Significance
		12 h	24 h	36 h	48 h	60 h	72 h	84 h	96 h				
L965	Dp	4	1	1	0	0	0	0	0	6	60.00	0.0043	*⁣* ^*∗∗*^
L966	Dp	10	0	0	0	0	0	0	0	10	100.00	>0.999	ns
SC42	Hp	9	0	0	0	0	0	0	0	9	90.00	0.3173	ns
Ss2301	P	10	0	0	0	0	0	0	0	10	100.00	>0.999	ns
Ss2401	P	10	0	0	0	0	0	0	0	10	100.00	>0.999	ns
P1/7	—	10	0	0	0	0	0	0	0	10	100.00	—	—
PBS	—	0	0	0	0	0	0	0	0	0	0	<0.0001	*⁣* ^*∗∗∗∗*^

*Note:* The survival curves of *S. suis* serotype 14 strains were compared with that of the highly pathogenic strain P1/7 using the Mantel–Cox test for statistical analysis. P indicates that the source was patients, Hp indicates that the source was healthy pigs, and Dp indicates that the source was diseased pigs. *⁣*^*∗∗*^, *⁣*^*∗∗∗∗*^, and “ns” indicate *p* < 0.01, *p* < 0.0001, and no significant difference, respectively.

## Data Availability

The data are available upon request from the authors.

## Supporting Information

Supporting Information 1 Table S1: The distribution of 55 putative virulence factors in serotype 14 *S. suis*.

Supporting Information 2 Table S2: The background information of 125 serotype 14 *S. suis* strains.

Supporting Information 3 Table S3: The background information of 318 ST7 *S. suis* strains.
